# *ANKS6* Variants Underlie Polycystic Kidneys in Prenatal and Neonatal Cases

**DOI:** 10.3390/genes15111374

**Published:** 2024-10-25

**Authors:** Lama S. Almohlesy, Faiqa Imtiaz, Maha Tulbah, Amal Alhashem, Manar Alhajooj, Abdullah Alhashem, Holly Mabillard, John A. Sayer, Khalid K. Alharbi, Mohamed H. Al-Hamed

**Affiliations:** 1 Pathology and Laboratory Medicine Department, King Faisal Specialist Hospital and Research Centre, P.O. Box 3354, Riyadh 11211, Saudi Arabia; lalmahlasi@kfshrc.edu.sa; 2 Department of Clinical Laboratory Sciences, College of Applied Medical Sciences, King Saud University, Riyadh 11433, Saudi Arabia; kharbi@ksu.edu.sa; 3 Centre for Genomic Medicine, King Faisal Specialist Hospital and Research Centre, MBC# 26, P.O. Box 3354, Riyadh 11211, Saudi Arabia; fahmad@kfshrc.edu.sa; 4 Department of Obstetrics and Genecology, King Faisal Specialist Hospital and Research Centre, P.O. Box 3354, Riyadh 11211, Saudi Arabia; mtulbah@kfshrc.edu.sa; 5 Prince Sultan Military Medical City, Riyadh 12233, Saudi Arabia; amal.alhashem@gmail.com (A.A.); aa-lhashem@psmmc.med.sa (A.A.); 6 College of Medicine and Medical Sciences, Arabian Gulf University, Manama P.O. Box 26671, Bahrain; manarmatarhajooj@hotmail.com; 7 Translational and Clinical Research Institute, Faculty of Medical Sciences, Newcastle University, Central Parkway, Newcastle upon Tyne NE1 3BZ, UK; holly.mabillard2@newcastle.ac.uk (H.M.); john.sayer@newcastle.ac.uk (J.A.S.); 8 National Institute for Health Research Newcastle Biomedical Research Centre, Newcastle upon Tyne NE4 5PL, UK; 9 Renal Services, Newcastle upon Tyne Hospitals NHS Foundation Trust, Newcastle upon Tyne NE7 7DN, UK

**Keywords:** nephronophthisis, *ANKS6*, whole-exome sequencing, echogenic kidney, consanguineous

## Abstract

Background: Nephronophthisis (NPHP) is an autosomal recessive genetic disorder that can cause early-onset kidney failure. *ANKS6* plays an important role in early kidney development and encodes a protein that interacts with other proteins within the primary cilium. *ANKS6* mutations are known to cause nephronophthisis 16 (NPHP-16). Little is known regarding fetal ultrasound imaging and the antenatal diagnosis of fetuses with *ANKS6*-associated kidney disease. Here, we report the detection of *ANKS6* variants in consanguineous families with polycystic kidney antenatally and in the early stages of life. Methods: Three unrelated Saudi Arabian patients (two prenatal patients and one neonate) were investigated. These cases were referred to the hospital due to the presence of echogenic kidneys on antenatal scanning. After clinical and phenotypic evaluation, whole-exome sequencing (WES) was performed on the cord and peripheral blood to identify the molecular genetic causes associated with the echogenic kidney phenotypes. Results: Two homozygous sequence variants were detected in *ANKS6*. The homozygous missense novel variant *ANKS6*: c.1159A>C was detected in Families 1 and 2. In the third family, the known homozygous loss-of-function variant *ANKS6*: c.907+2T>A was detected. Conclusions: We identified homozygous *ANKS6* variants in three families presenting with antenatal polycystic kidney disease. The findings provide an expanded clinical presentation of *ANKS6* and emphasize the utility of WES in the diagnosis of echogenic kidneys in prenatal settings.

## 1. Introduction

Cystic kidney disease phenotypes in childhood can be caused by a variety of diseases including non-hereditary kidney malformations or a range of inherited monogenic human diseases, including autosomal dominant and autosomal recessive polycystic kidney disease (PKD), nephronophthisis-related ciliopathies (NPHP), Meckel–Gruber Syndrome (MKS), and Bardet–Biedl Syndrome (BBS). Most of the early-onset cystic kidney diseases are associated with an autosomal recessive pattern of inheritance [1] and are termed renal ciliopathies.

NPHP is an autosomal recessive renal ciliopathy disorder responsible for 6–10% of kidney failure in children [2]. NPHP is clinically characterized by reduced urinary concentration and histologically by chronic tubulointerstitial fibrosis with corticomedullary cyst formation. It typically progresses to kidney failure before 30 years of age [2]. Three age-based clinical subtypes of NPHP are recognized, infantile, juvenile, and adolescent/adult, but molecular genetic diagnoses are more precise and allow for the investigation of associated extrarenal manifestations [3].

A new-born child with infantile NPHP may be affected by oligohydramnios that can lead to various complications, such as respiratory hypoplasia and facial dysmorphisms. In some cases, antenatal presentations of infantile NPHP may feature bilateral enlarged kidneys. These infantile NPHP presentations are associated with *INVS* and *NPHP3* mutations, but they have also been reported to be associated with *NEK8* and *CEP83* mutations [4]. Mutations in *INVS* can be associated with situs inversus or other laterality defects that are not typically seen in other forms of NPHP. Infantile NPHP is often associated with severe kidney dysfunction and rapid progression to kidney failure. It is important to recognize these early and atypical presentations which can mimic autosomal recessive PKD. More typical kidney ultrasound findings of NPHP also include increased echogenicity, reduced corticomedullary differentiation, and corticomedullary cysts. Sometimes, however, NPHP may present silently in childhood, and the decline in kidney function is often clinically silent and may present with established kidney failure [3].

In contrast to infantile forms of NPHP, most cases of NPHP are characterized by a normal or shrunken kidney size [3]. This phenotypic heterogeneity is interesting and is likely to be related to a profound disruption of primary ciliary signaling during the crucial early development of the kidney (and other organs) but is not yet fully explained. NPHP is genetically heterogenous, and mutations in more than 20 separate recessive genes have been reported [5].

The *ANKS6* gene is located on chromosome 9. It is additionally known as *PKDR1*, *SAMD6*, *NPHP16*, and *ANKRD14* [6]. Individuals with mutations in the *ANKS6* are prone to develop multiorgan ciliopathies, such as kidney cysts, visceral organ defects, and hepatic fibrosis. According to ClinVar Miner (https://clinvarminer.genetics.utah.edu/variants-by-gene/ANKS6/significance/pathogenic, accessed on 20 July 2024, there are 20 pathogenic variants identified in the *ANKS6* gene, and they are mainly associated with nephronophthisis 16. Genetic studies and cohort analyses have identified *ANKS6* mutations in patients with nephronophthisis, but no specific geographical prevalence has been established. The age presentation of NPHP associated with *ANKS6* variants is variable and has been reported in infants [7], children [8], and young adults [6].

The encoded ANKS6 protein is part of the ankyrin repeat family of proteins and localizes to the proximal region of the primary cilium known as the inversin compartment (INVc) where it regulates ciliary signaling [6,9]. The INVc is a periaxonemal subcompartment of the primary cilium, and to date, four proteins have been found to assemble within it, namely INVS, ANKS6, NEK8, and NPHP3. Mutations in the genes encoding the INVc proteins are known to cause various overlapping ciliopathy phenotypes in both animal models and humans, which include kidney defects and left–right asymmetry defects [6].

This paper describes three families who all had affected members presenting with echogenic kidneys in prenatal or early postnatal life with *ANKS6* disease causing variants. These cases provide examples in order to understand the complete disease phenotypic spectrum of *ANKS6*-associated kidney disease.

## 2. Materials and Methods

### 2.1. Human Subjects

**Figure 1:**
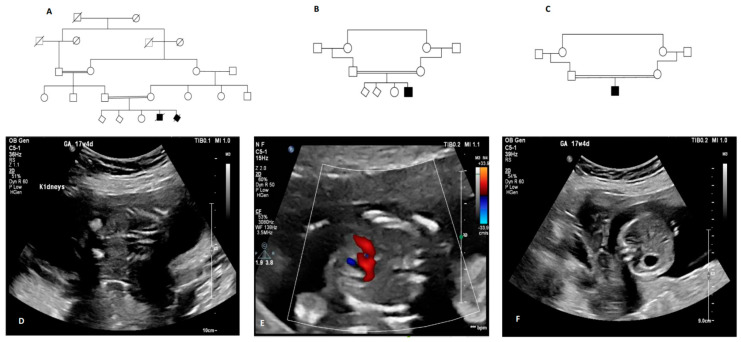
Pedigrees of participating families and fetal ultrasound of Family 1. (**A**) Pedigree diagram of Family 1. (**B**) Pedigree diagram of Family 2. (**C**) Pedigree diagram of Family 3. (**D**) Fetal ultrasound for Family 1 showing atrial septal defect. (**E**) Fetal ultrasound for Family 1 showing bilateral echogenic kidneys. (**F**) Fetal ultrasound for Family 1 showing situs inversus (stomach on right side).

We recruited three consanguineous unrelated Saudi Arabian families (Table 1, Figure 1), and echogenic kidneys were identified in the patients on prenatal or postnatal ultrasound scanning. Family and medical histories were recorded for all families.

This study was conducted according to the Declaration of Helsinki and approved by King Faisal Specialist Hospital and Research Centre (KFSH&RC) Ethics Committee (RAC# 2160 022). Written informed consent was collected from the probands’ parents. Specimens (fetal blood and peripheral blood) from fetuses, neonates, and their family members were collected for genomic DNA extraction. Genomic DNA was extracted using the Gentra Systems PUREGENE DNA Isolation kit (Qiagen, Germantown, MD, USA). Maternal cell contamination was ruled out using the AmpFLSTR^®^ Identifiler^®^ PCR Amplification Kit, as described by the manufacturer (Life Technologies, Warrington, UK).

### 2.2. Trio-WES and Variant Interpretation

For trio-WES, we used the same protocol as previously described [10]. In brief, genomic DNA from the index case and parents was used. The Illumina^®^ DNA Prep with Enrichment (Agilent Sureselect All Exons V6 (50 Mb) capture) kit was used to target the exon regions of their genomes. These targeted regions were sequenced using the NovaSeq 6000 sequencing system with an average target depth of 80×. The data of exomes were mapped to the human reference genome (NCBI build 37.1, UCSC hg19) using the Burrows–Wheeler Aligner (BWA) and Samtools. Variant calling was performed using the Genome Analysis Tool Kit (GATK) after the removal of duplicate reads by Picard. ANNOVAR was used to annotate all variants. For variant interpretation, an in-house variant interpretation pipeline was used to extend the public Annovar package. The in-house databases include collections of known disease-causing variants in the Saudi Arabian population and the aggregation of variants produced by samples from the Centre for Genomic Medicine (CGM-DB). Filtered variants were analyzed using QIAGEN Clinical Insight (QCI) Interpret. In addition, we used the DRAGEN copy number variant (CNV) pipeline calls of CNV events from next-generation sequencing (NGS) data, a reference-based normalization algorithm that uses additional matched normal samples to establish a baseline level from which to call CNV events. For variant sequence interpretation, candidate variants were prioritized according to phenotype. We prioritized rare non-synonymous and canonical splice site variants in Mendelian kidney disease identified using the OMIM database (https://www.omim.org/ accessed on 15 May 2024) and previously reported kidney genes [11]. We used the ACMG guidelines for variant classification [12]. The ClinVar and HGMD^®^ Professional 2023.2 databases were used to check for the novelty of the detected variants.

### 2.3. Sanger Sequencing

Sanger sequencing was performed to validate all detected variants. Primers for the PCR amplification of detected variants were designed using Primer3 software (http://frodo.wi.mit.edu/ accessed on 20 May 2024) and synthesized in-house. The amplified PCR products were sequenced using an ABI 3730xl capillary sequencer (Applied Biosystems, CA, USA), and the sequences were analyzed using Mutation Surveyor software V.3.24 (SoftGenetics LLC, State College, PA, USA).

### 2.4. Mutation Modeling

Alphafold sequence AF-Q68DC2-F1 was imported to PyMOL and labeled according to available UniProtKB data and the position of the missense SNV c.1159A>C; p.(Thr387Pro) highlighted in red (B). PyMOL Mutation Wizard was used to model the most likely structural impact of SNV c.1159A>C; p.(Thr387Pro) (C).

### 2.5. Blood Analyses

Biochemical investigations were performed in the biochemistry laboratory of Prince Sultan Military Medical City Hospital using standard commercial reagent kits.

## 3. Results

**Figure 2:**
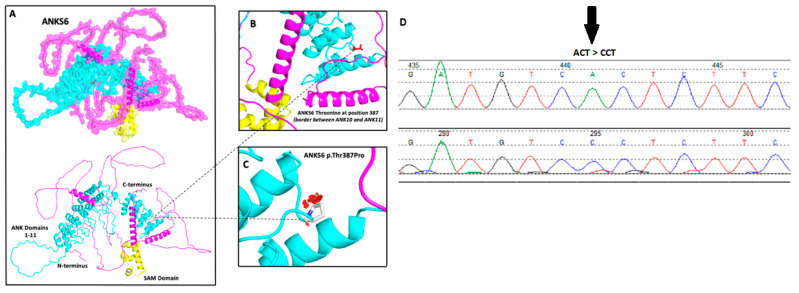
A sequence chromatogram of ANKS6 variant c.1159A>C and the modeling of ANKS6 p.Thr387Pro. (**A**) The predicted 3-dimensional structural model of human ankyrin repeat and sterile a motif (SAM) domain-containing six protein encoded by ANKS6 on chromosome 9, generated using the Alfafold Protein Structure Database (https://alphafold.ebi.ac.uk accessed on 21 May 2024) and UniProtKB (https://www.uniprot.org/uniprot/ accessed on 21 May 2024) with associated codes AF-Q68DC2-F1 and Q68DC2, respectively. The protein contains 11 ankyrin repeats (turquoise) starting near the protein’s N-terminus and a sterile a motif (SAM) (yellow) ending near the C-terminus. (**B**) The missense SNV c.1159A>C (p.Thr387Pro) position is highlighted in red at the border between ankyrin repeats 10 and 11. (**C**) Missense SNV c.1159A>C; p.(Thr387Pro) is modeled at the border between ankyrin repeats 10 and 11. The most probable rotamer (86% likelihood) is demonstrated. Considering mutational alteration SNV c.1159A>C; p.(Thr387Pro), the red discs represent the significant pairwise overlap of atomic van der Waals radii causing a likely structural ‘clash’. (**D**) A sequence chromatogram of ANKS6 homozygous missense variant NM_173551.5: c.1159A>C; p.(Thr387Pro).

In all three families enrolled, an echogenic kidney phenotype was observed in the affected child, suggesting an early-onset cystic kidney disease such as infantile NPHP. WES detected a likely molecular genetic diagnosis in all families. Molecular genetic variants were detected in *ANKS6*. Two affected fetuses (Families 1 and 2) had a homozygous missense variant *ANKS6*: NM_173551.5: c.1159A>C; p.(Thr387Pro). This novel variant is illustrated in Figure 2D. Using the Alphafold Protein Structure database, ANKS6 can be modeled in terms of its predicted 3-dimensional structure (Figure 2). ANKS6 is known to form an NPHP module protein complex. The ankyrin repeats within ANKS6 are necessary for NEK8 binding, which then connects to inversin (INVS) and NPHP3 to form a distinct NPHP module which is recruited to the proximal ciliary axoneme, forming the INVc [13]. ANKS6 also interacts via the SAM domain with BICC1 (via KH domains) in an RNA-dependent manner and with ANKS3 (via the SAM domain) [13,14]. The missense variant p.(Thr387Pro) occurs between the alpha helices of ankyrin 10 and ankyrin 11, part of the ANK domain necessary for NEK8 binding (Figure 2). In silico scores including the Combined annotation-dependent depletion score (CADD score) suggest that the amino acid substitution is damaging (Table 1).

In the child from Family 3, a homozygous splice variant *ANKS6*: NM_173551: c.907+2T>A was detected and was predicted to be pathogenic (Table 1). A detailed analysis of the clinical presentation and results for each family is given below.

**Family 1:** A 35-year-old pregnant women was referred to the KFSH&RC hospital with a diagnosis of an abnormal fetal ultrasound and a history of previous pregnancy losses. The couple consisted of first-degree relatives (Figure 1A). Her obstetric history showed that she had a first trimester abortion for both her first and second pregnancies. Her current (fourth) pregnancy (gestational age by fetal ultrasound (US) was 17 weeks + 4 days) showed abnormal echogenic kidneys bilaterally (Figure 1D). Other fetal abnormalities identified included congenital heart defects including an atrial and ventricular septum defect (Figure 1E) and situs inversus of the heart and the intestines (Figure 1F). Cord blood was obtained, and the pregnancy was terminated based on the antenatal US findings. Genetic studies following trio-WES revealed a homozygous missense variant in the fetus *ANKS6* (NM_173551.5): c.1159A>C; p.(Thr387Pro), and both parents were heterozygous for this allele (Figure 2D). Subsequently, another recent pregnancy of the family underwent prenatal diagnosis (PND) at 17 weeks gestation, and the homozygous missense variant c.1159A>C; p.(Thr387Pro) was found in the fetus, and echogenic kidneys were confirmed by fetal ultrasound.

**Family 2:** A 6-month-old baby boy from a consanguineous family (Figure 1B) was admitted to a local hospital. He had presented in the first days of his life with vomiting, resulting in dehydration and electrolyte disturbances. He was also found to be deeply jaundiced. His initial laboratory tests revealed raised liver enzymes and elevated serum creatinine levels (137 µmol/L) (Table 2).

A detailed abdominal ultrasound scan (USS) of the child showed that their liver was protruding with course echotexture but with no intrahepatic biliary dilatation and a normal appearance of the spleen. Renal USS revealed bilateral enlarged cystic kidneys. There was also significant ascites. The combination of findings suggested congenital hepatic fibrosis and an autosomal recessive PKD-like or infantile NPHP presentation. Genetic studies using trio-WES revealed a homozygous missense variant in *ANKS6* (NM_173551.5): c.1159A>C; p.(Thr387Pro). Parents and an unaffected sister were heterozygous for this allele. The child died at the age of 7 months secondary to progressive kidney disease and congenital hepatic fibrosis. Of note, Family 1 and Family 2 are both from the central region of Saudi Arabia, suggesting shared ancestry.

**Family 3:** A 28-year-old pregnant woman (gravida 1) was referred to the KFSH&RC hospital following antenatal scans showing enlarged fetal kidneys, anhydramnios, and cardiac anomalies including multiple ventricular septal defects (VSDs). The gestational age was 21 weeks + 1 day. The couple was known to be consanguineous (Figure 1C) but with no previous significant medical or family history. At 26 weeks gestation, a spontaneous vaginal delivery occurred, and the infant’s birth weight and height were 1190 g and 35 cm, respectively. The infant had multiple congenital anomalies: multiple VSDs, abnormal bilateral PKD, a deformed head with a prominent left temporal area, swollen eyelids, slight corneal opacity (both eyes), a flat nasal bridge, a bell-shape chest, flat nipples, a distended abdomen, organomegaly on the left side of the abdomen, significant bilateral talipes equinovarus deformity, and male genitalia. The infant survived for only a few hours before death. Trio-WES detected a homozygous splice variant in *ANKS6* (NM_173551.5): c.907+2T>A in the fetus, and both parents were heterozygous for this allele.

## 4. Discussion

As far as we know, this study is the first to describe *ANKS6*-associated disease phenotypes in a prenatal setting. It does agree with previous reports regarding the manifestation of the early onset of infantile NPHP with progressive cystic kidney disease leading to kidney failure [13]. All three cases shared phenotypes of enlarged cystic kidneys and severe extrarenal defects, including situs inversus and hepatic and cardiovascular abnormalities.

The homozygous missense variant in exon 5 of *ANKS6* (NM_173551.5): c.1159A>C; p.(Thr387Pro) was detected in two families, in prenatal and neonatal cases (Families 1 and 2). The variant is novel and was not found in a Saudi Arabian population database (CGM database) or in other public databases (gnomAD, 1000 Genomes Project, ESP). Familial segregation in the two consanguineous families confirmed the segregation of the variant and homozygosity by descent in the affected cases. The aggregated in silico prediction tools and conservation analysis predicted the uncertainty of the damage to the protein structure and function (Table 1). Indeed, the missense variant p.(Thr387Pro) occurred within the ANK domain necessary for ANKS6 and NEK8 binding. Previously, NEK8 binding as shown to be lost when the four N-terminal ANK repeats were deleted, while the truncation of only the first N-terminal ANK repeat resulted in only a minor reduction in NEK8 binding [9]. The missense variant is therefore in an important protein domain, but we do not know precisely what the implications are for this variant in terms of ANKS6 and NEK8 binding.

The splice variant in intron 3 of *ANKS6* (NM_173551): c.907+2T>A (rs1438673595; ClinVar: RCV001333047) was reported previously [8,15] and was predicted to cause a splicing defect with the subsequent loss of function. This variant was detected in Family 3 where the pregnancy ended with preterm delivery and early neonatal death. The main function of ANKS6 is to connect with and form a complex with NEK8, INVS, and NPHP3 [9]. The severe phenotypes seen in our *ANKS6* cases may be attributed to the location of variants and the resulting alteration to the composition of the ANKS6-INVS-NPHP3-NEK8 module. Both detected variants were located at the beginning of the gene in intron 3 (splice variant) and exon 5 (missense variant), and future in vivo experiments and a greater number of cases may prove this assumption. Unfortunately, our study did not include a pathological examination of kidney tissue samples, but USS appearances were consistent with an infantile NPHP picture.

This study provided all families with an accurate diagnosis that could be used to predict recurrence rates and allow them to plan for future pregnancies by performing prenatal diagnosis (PND) or Preimplantation Genetic Testing (PGT). Indeed, Family 1 benefited from a PND approach, and the affected pregnancy was terminated in the early stages because of the detection of the homozygous *ANKS6* variant.

The findings of this study support the application of trio whole-exome sequencing in prenatal/neonatal diagnosis as a routine test. This approach can enhance the detection of rare Mendelian disorders and contribute to the prevention and management of pregnant women at risk, especially in highly consanguineous populations.

## 5. Conclusions

Our findings highlight the importance of ANKS6 in the development of the kidney, liver, and heart and the associated primary ciliopathy phenotypes seen with disease-causing variants. This study presents the important contribution of *ANKS6* in early-onset cystic kidney disease and infantile NPHP as well as other multisystem features consistent with its role in the primary cilium.

## Figures and Tables

**Table 1:** Genetic variants detected in ANKS6 and in silico analysis of pathogenicity.

Family #	Family History	No. of Affected (Suspected)	Clinical Phenotype	Outcome	HGVS (hg38)	Nucleotide Change (NM_173551.5)	Amino Acid Change (Q68DC2)	Zygosity	PolyPhen-2 (Score)	SIFT (Score)	CADD	ACMG Classification
Family 1	Yes	2	Antenatal presentation (17 weeks gestation) Bilateral echogenic kidneys Congenital heart defects Situs inversus of the heart and the intestines	Termination of pregnancy at 17 weeks	chr9:g.98782527T>G	*ANKS6*: c.1159A>C	p.Thr387Pro	Homozygous	Possibly Damaging (0.917)	Damaging (0.011)	23.1	VUS (PM1, PM2)
Family 2	Yes	2	Neonatal presentation Bilateral enlarged cystic kidneys Congenital hepatic fibrosis Ascites, Cholestasis Systemic hypertension Portal hypertension	Infant death at 7 months of age	chr9:g.98782527T>G	*ANKS6*: c.1159A>C	p.Thr387Pro	Homozygous	Possibly Damaging (0.917)	Damaging (0.011)	23.1	VUS (PM1, PM2)
Family 3	Unknown	None	Antenatal presentation (21 weeks gestation) Bilateral enlarged cystic kidneys Multiple congenital anomalies Distended abdomen Bilateral talipes equinovarus deformity	Neonatal death (1 day)	chr9:g.98784830A>T	*ANKS6*: c.907+2T>A	p.?	Homozygous	N/A	N/A	32	Pathogenic (PVS1, PM2, PP3, PP5)

**Table 2:** Biochemical findings in index case of Family 2.

Test	Results	Normal Range	Indication
**Full Blood Count**			
WBC	10.1	6–18 × 10^9^/L	
RBC	2.79 L	4.1–5.3 × 10^12^/L	Low
HGB	7.6 L g/dL	111–141 g/L	Low
HCT	0.231%	0.300–0.400%	Low
MCV	83 fL	68–84 fL	
MCH	27.2 pg	24–30 pg	
MCHC	32.9 g/dL	300–360 g/L	Low
RDW	18.4%	11.0–15.0%	High
PLT	266	200–550 × 10^9^/L	
MPV	10.5 fL	6.3–11.2 fL	
**Serum Electrolytes**			
Sodium	145 mmol/L	135–147 mmol/L	
Potassium	5.3 mmol/L	3.7–5.9 mmol/L	High
Bicarbonate HC03	28.1 mmol/L	16–24 mmol/L	High
Urea	33.8 mmol/L	1.5–6.8 mmol/L	High
Creatinine	137 µmol/L	26–62 µmol/L	High
Albumin	39 g/L	37–49 g/L	
Calcium	2.55 mmol/L	2.10–2.60 mmol/L	
Corrected Calcium	2.58 mmol/L	2.2–2.7 mmol/L	High
**Liver Function Tests and Coagulation Studies**			
Alkaline Phosphatase (AP)	456 U/L	122–469 U/L	High
Total Bilirubin	354 µmol/L	0–21 µmol/L	High
Direct Bilirubin	327 µmol/L	0–5.0 µmol/L	High
Gamma GT (GGT)	180 U/L	11–49 U/L	High
Alanine Transaminase (ALT)	75 U/L	10–45 U/L	High
Aspartate Transaminase (AST)	56 U/L	10–45 U/L	High
Prothrombin Time (PT)	16.0 s	12.3–14.2 s	High
Activated Partial Thromboplastin Time (aPTT)	52 s	30.5–40.4 s	High

## Data Availability

All data generated during this study are included in this published article.
